# Hypothalamic Gene Expression and Postpartum Behavior in a Genetic Rat Model of Depression

**DOI:** 10.3389/fnbeh.2020.589967

**Published:** 2020-10-22

**Authors:** Wendy Luo, Patrick H. Lim, Stephanie L. Wert, Stephanie A. Gacek, Hao Chen, Eva E. Redei

**Affiliations:** ^1^Department of Psychiatry and Behavioral Sciences, Feinberg School of Medicine, Chicago, IL, United States; ^2^Department of Pharmacology, Addiction Science, and Toxicology, University of Tennessee Health Science Center, Memphis, TN, United States

**Keywords:** Wistar Kyoto More Immobile, oxytocin receptor, vasopressin, vasopressin receptor 1a, lysine demethylase 5A, period 2

## Abstract

Postpartum depression is a complex illness that often occurs in genetically predisposed individuals. Closely related inbred rat strains are a great resource to identify novel causative genes and mechanisms underlying complex traits such as postpartum behavior. We report differences in these behaviors between the inbred depression model, Wistar Kyoto (WKY) More Immobile (WMI), and the isogenic control Wistar Kyoto Less Immobile (WLI) dams. WMI dams showed significantly lower litter survival rate and frequency of arched back and blanket nursing, but increased pup-directed licking, grooming, and retrieval during postpartum days (PPD) 1–10, compared to control WLIs. This increased pup-directed behavior and the frequency of self-directed behaviors segregated during selective breeding of the progenitor strain of WKY, which is also a depression model. These behaviors are manifested in the WMIs in contrast to those of WLIs. Furthermore, habitual differences in the self-directed behavior between light and dark cycles present in WLIs were missing in WMI dams. Hypothalamic transcript levels of the circadian rhythm-related gene Lysine Demethylase 5A (*Kdm5a*), period 2 (*Per2*), and the maternal behavior-related oxytocin receptor (*Oxtr*), vasopressin (*Avp*), and vasopressin receptor 1a (*Avpr1a*) were significantly greater in the post-weaning WMI dams at PPD 24 compared to those of WLIs, and also to those of WMI dams whose litter died before PPD 5. Expression correlation amongst genes differed in WLI and WMI dams and between the two time-points postpartum, suggesting genetic and litter-survival differences between these strains affect transcript levels. These data demonstrate that the genetically close, but behaviorally disparate WMI and WLI strains would be suitable for investigating the underlying genetic basis of postpartum behavior.

## Introduction

Maternal behavior has long-term effects on the brain development of offspring, and depressive disorders impair maternal behaviors. One of the largest risk factors for depressive episodes in the perinatal period is depression before pregnancy (Rich-Edwards et al., 2006; Grant et al., 2008; Topiwala et al., 2012; Perani and Slattery, 2014). While most animal models of postpartum depression focus on mirroring the group of women who are experiencing depression for the first time in their life during postpartum (Perani and Slattery, 2014; Putnam et al., 2017; Eid et al., 2019), the present study employs a genetic model of depression-like behavior and its isogenic control strain to begin to investigate characteristics of these dams during postpartum, modeling the risk factor of depression before pregnancy.

The genetic rat model of depression-like behaviors, the Wistar Kyoto (WKY) More Immobile (WMI) rat strain, was bi-directionally selectively bred from the parental WKY strain. The WKY rat strain is a well-established model for depression as its behavior mirrors symptoms of human major depression and anxiety, including despair-like behavior, excessive anxiety, learned helplessness, disturbed sleep patterns, and hypoactivity (Paré and Redei, 1993; Paré, 1994a,b; Dugovic et al., 2000; Redei et al., 2001; Solberg et al., 2001, 2004; Malkesman et al., 2005; Baum et al., 2006). Chronic treatments with antidepressants, electroshock administration (model for electroconvulsive therapy), and deep-brain stimulation can all reverse these depression-like behaviors (Jeannotte et al., 2008; Falowski et al., 2011; Kyeremanteng et al., 2012). The WKY strain was developed as the normotensive control for the Spontaneously Hypertensive Rat (SHR) strain. Louis and Howes (Louis and Howes, 1990) demonstrated that the WKY strain was distributed to different vendors and universities between F12 and F17 generations of inbreeding.

The fact that the WKY rats showed genetic and behavioral differences (Kurtz et al., 1989; Paré and Kluczynski, 1997) motivated the bi-directional selective breeding using FST immobility as a functional selector (Will et al., 2003). The WMIs, now at their 44^th^ generation and completely inbred after >35 generations of full-sib breeding, show despair-like behaviors and greater sensitivity to stress compared to their isogenic control counterparts, the WKY Less Immobile (WLI) rats (Will et al., 2003; Andrus et al., 2012; Lim et al., 2018b). While the WMIs show higher immobility behavior in the forced swim test, which was the original functional selector for this strain, WLI males and females present immobility behavior comparable to that of other control strains. In our lab, Sprague–Dawley and Fischer 344 male rats show immobility very similar to that of WLI males (Solberg et al., 2003; Wilcoxon et al., 2005; Andrus et al., 2012; Mehta et al., 2013; Mehta-Raghavan et al., 2016). WLI female immobility is similar to that of Wistar and F344 females (Kokras et al., 2018). Through the WMI genetic model of depression and its WLI control strain, any observed behavioral and transcriptomic differences may be directly or indirectly related to the identified <5,000 sequence variations between the two strains (Chen et al., 2017; Bryant et al., 2020).

During the early postnatal period, adaptive changes occur in the mothers’ (dam) neuroendocrine system, which enables them to provide appropriate maternal care. These adaptive changes are pivotal and brought about by the hormonal alterations due to parturition (Levy, 2016). The decrease in estrogen and/or progesterone at parturition may directly affect maternal behaviors (Hauser and Gandelman, 1985; Glynn et al., 2016; Murakami, 2016). Changes in estrogen levels alter the expression of its receptors with subsequent effects on the transcription of their target genes and their receptors. Clinical trials involving estrogen as a potential treatment for negative maternal behaviors are ongoing; for example, one study investigated the potential use of transdermal estradiol as a treatment for postpartum depression (Wisner et al., 2015). In addition to hormonal regulation, several genes and their pathways have been shown to influence maternal behavior, including those related to oxytocin (*Oxt*) and vasopressin (*Avp*). Oxytocin is released within various brain regions including the paraventricular nucleus (PVN), supraoptic nucleus, septum, hippocampus, and olfactory bulb (Bosch and Neumann, 2012). In humans, mothers with higher oxytocin expression show increased maternal touch and contact with their children (Pratt et al., 2015). In rats, Oxt release leads to mothers fostering more positive interactions with their offspring (Leng et al., 2008), and central administration of Oxt receptor antagonist can block the onset of maternal care, and reduce pup-directed behaviors (Champagne et al., 2001; Pedersen and Boccia, 2003). The medial preoptic area seems to be a key brain region of vasopressin (Avp) actions on maternal care. It receives vasopressinergic input from the suprachiasmatic nucleus, and diurnal changes in local Avp release have been described (Kalsbeek and Buijs, 2002). Vasopressin regulates maternal care and it seems to occur *via* vasopressin receptor 1a (Avpr1a; Pedersen et al., 1994; Bosch et al., 2007, 2010). As many of the Oxt- and Avp-relevant brain regions are within the hypothalamus, which is also intimately involved in affecting maternal behaviors (Fang et al., 2018), we focused on the expression of these neuropeptides and their receptors in the whole hypothalamus in this study.

The purpose of this study is to determine whether a genetic model of depression, the WMI rat, shows alterations in maternal functioning compared to its isogenic control strain. Here, we explore postpartum behaviors and expression of relevant hypothalamic genes in these strains of inbred rats.

## Materials and Methods

### Animals and Behavioral Assessments

All animal procedures were approved by the Institutional Animal Care and Use Committee of Northwestern University. The 40th generation WLI and WMI inbred strains were housed under temperature and humidity-control with food and water *ad libitum* on a 12:12 LD cycle, lights on at 06:00 h. During the experiment, red lights were on between 18:00 and 23:00 h to allow video recording during the dark phase.

Females of both strains (18 for WLI and 28 for WMI) were mated for this study. Maternal behavior of dams (six WLI and six WMI) that birthed litters that survived to wean (postpartum day 24: PPD 24) was monitored and recorded daily. Maternal behavior was observed for 10 days PPD 1–PPD 10, as described before (Ahmadiyeh et al., 2004), which was a modification of previous studies (Myers et al., 1989; Francis et al., 2003). Behaviors were automatically recorded for an hour under light (11:00–14:00 h) and dark (18:00–23:00 h) conditions, starting at PPD 1, the day after birth. Behavioral analyses were scored manually. Blind scoring was not feasible as the WMI strain had many more mating pairs (to account for litter loss) and, therefore, the experimenter was aware of which strain was giving birth at any given time. The following behaviors were scored every 3 min: arched-back nursing; blanket nursing (mother lies over pups); proximity to pups, which is either passive nursing (mother is on the side or back with pups feeding) or just resting with pups very close by; licking/grooming of pups and pup retrieval; no contact (mother leaves pup alone more than half a cage length away); or self-directed behaviors (eating and drinking; Ahmadiyeh et al., 2004). Arched back nursing and blanket nursing categories were combined, and also licking/grooming of pups and pup retrieval behaviors. Behavioral measures were shown as a frequency of observations for each hour of monitoring.

Litter deaths were observed during a previous 8-month mating period, and results from this period prompted the investigation of maternal behavior in this study. Litter death was also recorded in the current study. Although we have not made a quantitative assessment, most pups that died before weaning either died from cannibalism by the mother or showed signs of undernourishment with no milk in their stomachs.

Dams were euthanized, either before PPD 5 after their litters died (four WLI and six WMI) or at PPD 24 (six WLI and six WMI) after the pups were weaned, during lights on at 11:00–14:00 h by fast decapitation.

### Brain Dissection and RNA Isolation

Hypothalami were dissected with a brain matrix according to Paxinos coordinates (anterior-posterior, −0.30 to −4.16; medial-lateral, 0–2.2; dorsal-ventral, −0.40 to −2.8) and were temporarily stored in RNAlater (Ambion, Austin, TX, USA) at −80°C. Tissue samples were homogenized using a TRIzol reagent (Ambion, Austin, TX, USA) and total RNA from each hypothalamic sample was isolated with Direct-zol RNA MiniPrep Kit (Zymo Research, Irvine, CA, USA) following the manufacturer’s protocol. Once isolated, 1 μg of the total RNA was reverse transcribed using the SuperScript VILO cDNA Synthesis Kit (Thermo Fisher Scientific, Waltham, MA, USA). All of these methods have been described previously (Raghavan et al., 2017; Lim et al., 2018a,b; Meckes et al., 2018).

### Real-Time Reverse Transcription-Polymerase Chain Reaction (RT-qPCR)

For each experimental group, RT-qPCR was performed to compare the hypothalamic target gene expression levels between strains (WLI vs. WMI). Primers for each target gene were designed using Applied Biosystems Primer Express software (version 3.0, PE Applied Biosystems, Foster City, CA, USA); the primer sequences can be found in Supplementary Table 1. Five ng of cDNA was amplified in a 20 μl reaction using SYBR Green Master Mix (Thermo Fisher Scientific, Waltham, MA, USA) in the QuantStudio 6 Flex Real-Time PCR System (Thermo Fisher Scientific, Waltham, MA, USA). Triplicates of reactions were performed and reached threshold amplification within 34 cycles. Target transcript levels were quantified relative to Gapdh, a housekeeping gene previously demonstrated to show similar expression across strains and conditions, and to a general WLI male hypothalamic calibrator using the 2^(−ΔΔCT)^ method.

### Statistical Analysis

Data are presented as mean ± standard error of the mean. All statistical analyses were performed using GraphPad Prism v8.0 (GraphPad Software, La Jolla, CA, USA). Behavioral observations were analyzed across postpartum days for the light and the dark phase by two-way ANOVA with repeated measures or mixed effect models, followed by Sidak’s *post hoc* analysis for multiple comparisons. Cohen’s *d* values were calculated by using the Cohen’s *d* = (M2 − M1)/SD_pooled_ equation, where M1 and M2 are means of the groups to be compared. Gene expression differences were analyzed by two-way ANOVA followed by Tukey’s multiple comparison test. Pearson correlation of the gene expression data was corrected for multiple comparisons. Technical outliers, when multiple days of behavioral observations were lost or when RT-qPCR data were marked by the program as technical outliers, were omitted from the analysis.

ANOVAs, mixed effect analyses, and Cohen’s *d* effect sizes are described in the results, while *post hoc* analyses are shown on the figures.

## Results

### Litter Statistics

Body weights of adult female WLIs were significantly higher compared to those of same age WMI females before mating (175.3 ± 2.3 g vs. 134.7 ± 1.6 g; *p* = 1.9e-15).

WLI and WMI litters differed significantly in their rate of survival to weaning (*p* = 0.044; Table 1). While the WLI litters had a survival rate of 50%, their WMI counterparts had a survival rate of less than half, 21.4%. This survival rate is in agreement with the one observed during a previous 8-month mating period (13 WLI litters survived out of 22 total litters for a 59% rate compared to 10 WMI litters survived from a total of 37 litters, 27% survival rate; *p* = 0.014). Additionally, WMI litters seemed to be smaller at birth compared to WLI litters, based on the counts without disturbing the cage. Due to the high pup mortality, the exact number of pups at birth was not determined since we did not want to disturb the dams and litters after birth. WMI litters had significantly fewer pups than the WLI litters at weaning (*t*_(22)_ = 3.13, *p* = 0.005; Cohen’s *d* = 1.28), and the numbers were uneven between the sexes with less male WMI pups survived compared to WLI males (males, *t*_(22)_ = 2.29, *p* = 0.036; Cohen’s *d* = 0.91; females, *t*_(22)_ = 1.82, *p* = 0.082; Cohen’s *d* = 0.74; Table 1).

**Table 1:** Litter characteristics.

Strain	Litters survived/total; % survival	Days survived by litters not weaned	Number of pups at weaning	Male : female ratio at weaning
WLI	9/18, 50%	5.22 ± 2.17	8.50 ± 0.68	4.41 ± 0.55 : 4.08 ± 0.41
WMI	6/28*, 21.4%	3.375 ± 0.63	5.92 ± 0.47**	2.83 ± 0.44*: 3.08 ± 0.35

Values are mean ± SEM; *p < 0.05, **p < 0.01 strain differences.

### Maternal Behavior

**Figure 1:**
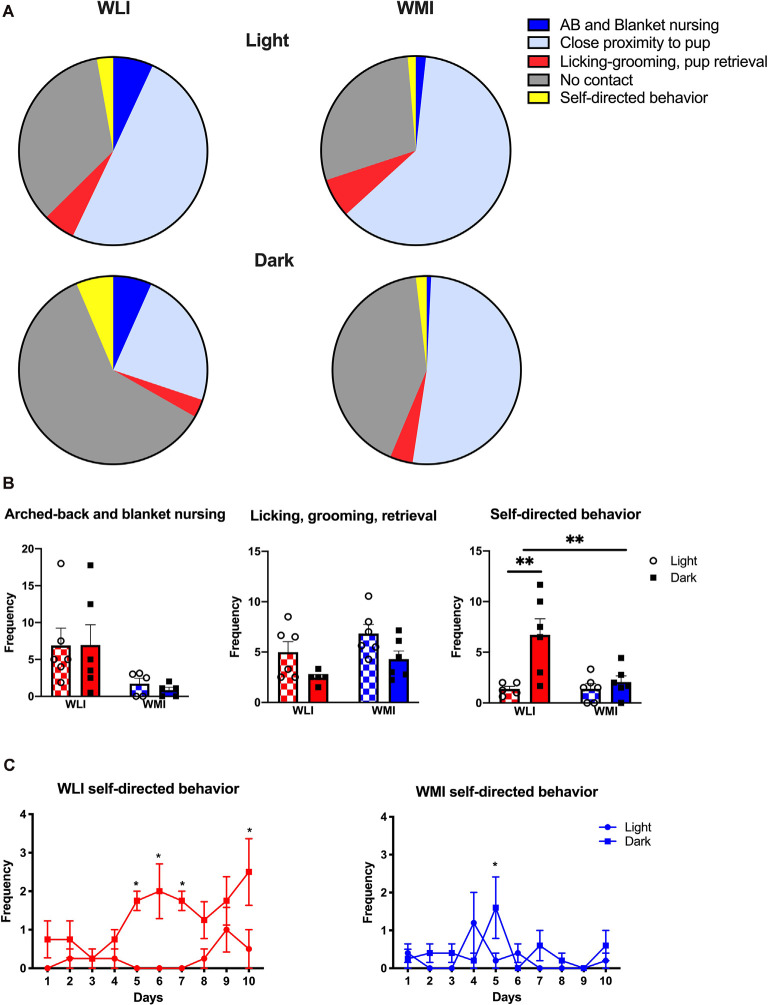
Maternal and self-directed behaviors of Wistar Kyoto Less Immobile (WLI) and Wistar Kyoto More Immobile (WMI) dams as observed during postpartum day 1–10. **(A)** Percentage of average frequency/hr of each category of maternal behaviors in the light and the dark phases of the day are shown. **(B)** Frequency per hour of observation of arched-back and blanket nursing is greater in WLI dams compared to WMIs, regardless of the time of day. Pup-directed behaviors, such as licking, grooming, and retrieval are greater in WMI than WLI dams and differ between the light and the dark phases of the day, the latter being less. Self-directed behaviors do not differ between WLI and WMI dams during the light phase, but during the dark phase (when nocturnal animals are more active), WMI dams spend significantly less time eating and drinking compared to WLIs. **(C)** Self-directed behaviors are greater in the dark phase in WLIs across the 10 days observation, with only one time of day difference in WMIs. Data are presented as mean ± standard error of the mean. *p < 0.05, **p < 0.01.

Maternal and self-directed behaviors were assessed for 10 days postpartum and their distribution is shown in Figure 1A. The figure shows the percentage of different behaviors in both strains, measured as the average frequency observed during the 1-h observation period in the light and the dark phase. A significant strain difference was observed in arched-back and blanket nursing (strain, *F*_(1,18)_ = 7.77, *p* = 0.012), with no differences between light and dark phases (Figure 1B). Large effect sizes were detected for the strain comparisons (Cohen’s *d*, light: 1.23; dark: 1.28). Similarly, there were strain differences in licking, grooming, and pup retrieval (*F*_(1,19)_ = 4.61, *p* = 0.045; Figure 1B), with WMIs showing the greater of these pup-directed behaviors. The effect sizes revealed that this strain difference originated more from the dark phase comparison (Cohen’s *d*, light: 0.76; dark: 1.24). Both strains of dams showed a lower frequency of these pup-directed behaviors during the dark phase (time of day, *F*_(1,19)_ = 8.79, *p* = 0.008). Self-directed behaviors such as eating, drinking, and self-grooming, also differed by strain and time of day (strain, *F*_(1,19)_ = 6.12, *p* = 0.023; time of day *F*_(1,19)_ = 10.29, *p* = 0.005; Figure 1B). Interestingly, the diurnal change in this behavior was only seen in the WLI dams (strain × time of day, *F*_(1,19)_ = 6.19, *p* = 0.022). This is confirmed by the large effect size in strain comparisons at the dark phase (Cohen’s *d*, light: 0.02; dark: 1.59).

Figure 1C shows self-directed behaviors across the 10 days observation period for both WLI and WMI dams. WLI dams showed day by day differences in self-directed behaviors across the observation period, and also between the light and dark phases (time of day, *F*_(1,3)_ = 23.42, *p* = 0.017; days × time of day, *F*_(9,27)_ = 2.45, *p* = 0.035). Specifically, self-directed behaviors were significantly greater in the dark phase than the light phase on PPD 5–7 and 10 (PPD 5, *t*_(30)_ = 3.56, *p* = 0.013; PPD 6, *t*_(30)_ = 4.07, *p* = 0.003; PPD 7, *t*_(30)_ = 3.56, *p* = 0.013, and PPD 10, *t*_(30)_ = 4.07, *p* = 0.003). In contrast, there were no significant differences in self-directed behaviors across the days of observation in WMIs, but light, dark phase differences tended to occur postpartum day 5 (days × time of day, *F*_(9,35)_ = 2.12, *p* = 0.054; PPD 5, *t*_(39)_ = 3.07, *p* = 0.038).

### Hypothalamic Target Gene Expression

**Figure 2:**
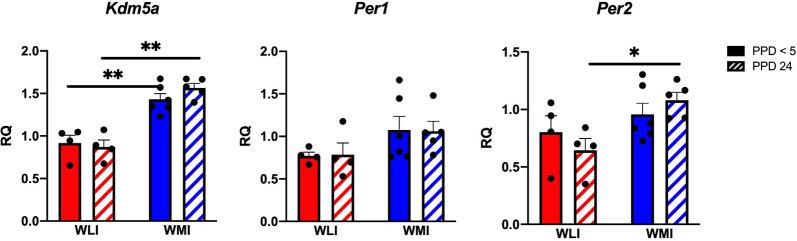
Hypothalamic gene expression of *Kdm5a*, *Per1*, and *Per2* in WLI and WMI dams who lost their litter before postpartum day 5 (PPD < 5) and in those whose litter were weaned at PPD 24. Hypothalamic transcript levels were measured by quantitative RT-PCR and shown as Relative Quantification (RQ). Data are presented as mean ± standard error of the mean. *N* = 4–6 per group. *P < 0.05 and **P < 0.01 represent *post hoc* comparisons.

The observation of the lack of circadian rhythm in the self-directed behaviors of WMI dams prompted us to examine any potential causative genetic differences in circadian rhythm-regulating genes between WLI and WMI. The Lysine Demethylase 5A (*Kdm5a* or Jumonji/ARID domain-containing protein1A: *JARID1A*) is connected to the circadian epigenome (Masri and Sassone-Corsi, 2013). We first identified the Arg745Cys variation in the *Kdm5a* gene between WMI and WLI strains from whole-genome sequencing data. This was validated using Sanger Sequencing (Supplementary Figure 1). Therefore, the hypothalamic expression of *Kdm5a* and the transcribed clock genes period 1 and 2 (*Per1*, *Per2*) was measured. Transcript levels of *Kdm5a* differed significantly between WLI and WMI females, regardless of postpartum days (*Kdm5a*, *F*_(1,15)_ = 68.77, *p* < 0.0001), with WMI dams showing higher expression (Figure 2). Transcript levels of *Per1* and *Per2* also differed significantly between WLI and WMI females (*Per1*, *F*_(1,15)_ = 4.63, *p* = 0.048; *Per2*, *F*_(1,15)_ = 8.05, *p* = 0.013), with WMI dams showing higher expression (Figure 2).

**Figure 3:**
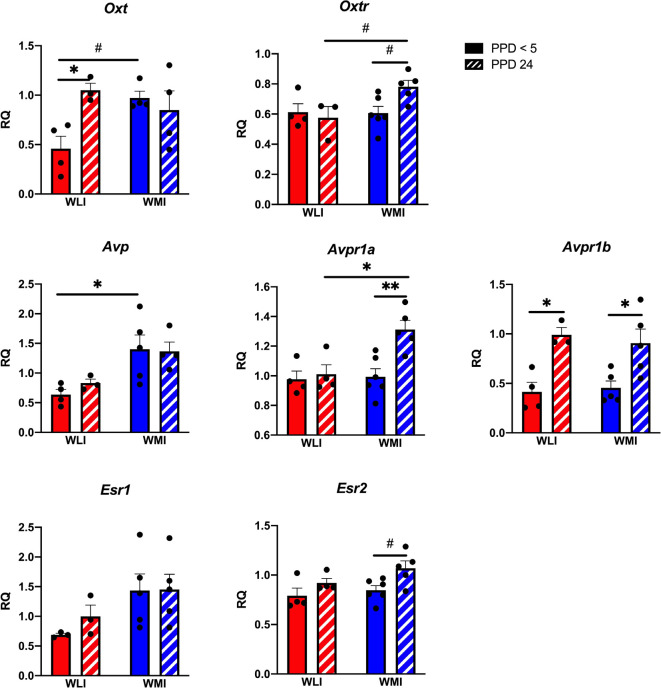
Hypothalamic gene expression of *Oxt, Oxtr, Avp, Avpr1a and Avpr1b, and Esr1 and Esr2* in WLI and WMI dams who lost their litter before postpartum day 5 (PPD < 5) and in those whose litter were weaned at PPD 24. Hypothalamic transcript levels were measured by quantitative RT-PCR and shown as Relative Quantification (RQ). Data are presented as mean ± standard error of the mean. *N* = 4–6 per group. ^#^< 0.10, *P < 0.05, and **P < 0.01 represent *post hoc* comparisons.

Hypothalamic transcript levels of target genes are shown in Figure 3. Interestingly, while *Oxt* expression tended to differ significantly between WLI and WMI dams only by litter survival (litter survival by strain, *F*_(1,11)_ = 7, 15, *p* = 0.022), expression of *Oxtr* showed both a clear strain and a strain by litter survival effect (strain, *F*_(1,13)_ = 4.80, *p* = 0.047; litter survival by strain, *F*_(1,13)_ = 5.38, *p* = 0.037). The *Avp* system showed major differences between the strains and litter survival. Specifically, expression of both *Avp* and *Avpr1a* showed strain differences (*Avp, F*_(1,12)_ = 12.54, *p* = 0.004; *Avpr1a, F*_(1,15)_ = 7.01, *p* = 0.018). However, while there were no litter survival and strain by litter survival effects for *Avp* expression, *Avpr1a* expression was greater in PPD 24 WMI hypothalamus compared to all other groups (litter survival, *F*_(1,15)_ = 8.64, *p* = 0.010; strain by litter survival*, F*_(1,15)_ = 5.62, *p* = 0.032). In contrast, hypothalamic transcript levels of *Avpr1b* did not differ between the strains but showed a significant association with litter survival (*F*_(1,13)_ = 21.56, *p* = 0.0005). Expression of *Esr1* differed significantly between the WLI and WMI hypothalamus (*F*_(1,12)_ = 5.24, *p* = 0.041), but the expression of *Esr2* only showed a difference by litter survival (*F*_(1,15)_ = 7.97, *p* < 0.013).

Heatmaps of the correlations between hypothalamic gene expressions are shown in Supplementary Figure 2 for WLIs and WMIs. The heatmaps illustrate the differential pattern of correlations between the strains and between the days postpartum. Unique strain differences in a significant correlation are present in WLIs regardless of PPD, such as the correlation between hypothalamic expression of *Oxtr* and *Avpr1a* and *Kdm5a* and *Per2*. These correlations are not present in WMIs, while the *Avp*, *Per1*, *Kdm5a*, and *Avpr1b* correlations are unique to PPD 24 WMI hypothalamus. Correlations unique to postpartum days or litter survival regardless of strain indicate that in the PPD <5 groups, hypothalamic expression of *Oxt* correlated with *Avp*, while at PPD 24, *Avpr1a* correlated with *Esr2* expression.

## Discussion

The WMI genetic animal model of depression has shown major differences during postpartum compared to their isogenic controls, the WLI strain that does not indicate depression-like behavior. Many of these characteristics segregated from the WKY parent phenotype during selective breeding with most behavioral phenotypes of WMIs being the same as the WKYs, while WLIs being different. Particularly, WMI dams have reduced litter survival, smaller litter size and decreased arched-back and blanket nursing, but increased licking, grooming retrieval behaviors toward their pups than WLI dams over the first 10 days postpartum. WMIs self-directed behaviors are decreased and WMIs show no diurnal rhythm in these behaviors while WLI dams do. This is similar to the biological rhythm disturbances observed in patients with depression compared to healthy controls (Mondin et al., 2017; Ozcelik and Sahbaz, 2020). Hypothalamic expression of *Kdm5a*, *Per1*, and *Per2* is greater in WMIs than WLIs, both in dams whose litter died before postpartum day 5 and in those whose litter survived to wean. Hypothalamic transcript levels of *Oxtr*, *Avp*, and *Avpr1a* are also increased in the WMI dams compared to their isogenic control WLIs, but only in dams whose litter survived to wean. Another parallel to the human condition is that these gene expression increases are similar to what was found in the hypothalamus of depressed patients (Meynen et al., 2006; Wang et al., 2008). Since WMI dams show characteristics similar to depressed patients and decreased nursing, but increased pup-directed behaviors, we propose that the dams of the WMI genetic animal model of depression are a potential animal model of postpartum depression.

In prior studies, differences in maternal care were not related to basic measures of reproductive success, such as litter survival to weaning (Champagne et al., 2003). The parental strain of the WLI and WMI, the WKY rat, has an average litter size of 8.16 pups which is similar to litter sizes of the control WLI dams and other rat strains (Gill Iii et al., 1979). However, after the establishment of these new inbred strains, the litter size for the WMI dams became close to half of that of the WLI dams. Furthermore, although WMI litters were birthed at the same rate as WLI litters, they showed a significantly lower survival rate than WLI litters. One of the potential causes of these findings could be aberrant maternal behavior of the WMI dam, as more nourishment-directed maternal behaviors are thought to increase litter survival (Weber et al., 2016). While dams of both strains show limited arched-back and blanket nursing behaviors during the observation period, WMI dams spent significantly less time with arched-back and blanket nursing of the pups than WLIs. In contrast, WMI dams spent more time licking, grooming, and retrieval of the pups than WLIs. Although arched-back, blanket nursing and licking, grooming and retrieval of the pups often co-occur, previous studies have found them to vary independently. For example, undernourished pups elicit increased licking, grooming from dams regardless of the dam’s ability or frequency of nursing (Lynch, 1976). It has been suggested that these two behaviors have distinct developmental roles with distinct effects on offspring biobehavioral outcomes, and also that arched-back nursing is typically not a significant predictor of offspring outcomes (Jensen Peña and Champagne, 2013).

Altered maternal-child interactions are reported when women suffer from postpartum depression and comorbid anxiety. Decreased breastfeeding, or premature cessation of it, has been observed in women with depression before or during pregnancy (Wallenborn et al., 2018; Jordan et al., 2019). Genetic rodent models with depression-like behavior show some of the characteristics of these altered interactions, as seen with the decreased nursing of WMIs compared to WLIs. Although maternal behavior has been examined in many inbred and outbred strains of rats and mice (Perani and Slattery, 2014), findings from the Flinders Sensitive Line (FSL) and the WKY rats, two different genetic animal models of depression (Lavi-Avnon et al., 2005; Braw et al., 2009), are particularly relevant to the current study. FSL dams do not differ from control Sprague Dawley dams in their maternal behavior, while WKY dams perform more pup-directed activities and fewer self-directed activities compared to Wistar controls (Braw et al., 2009). This again implies that the WKY parental phenotype of pup-directed and self-directed activities are segregated together and present in the WMI strain, differently from that of the litter size phenotype. Interestingly, increased pup-directed behavior could represent the “helicopter parenting” maternal phenotype observed in mothers with postpartum depression and anxiety (Perani and Slattery, 2014). Even more specifically, maternal anxiety is associated with higher maternal control and intrusiveness in the mother-infant interaction (Stein et al., 2012; Parfitt et al., 2013; Hakanen et al., 2019). Since both WKY (Solberg et al., 2003; Baum et al., 2006) and WMI females show concurrent increased depression and anxiety-like phenotypes (Mehta et al., 2013) and more pup-directed activities compared to their respective controls (Wistar and WLI; Braw et al., 2009), it is feasible that their behavior is also associated with maternal anxiety. This is similar to findings in which women with postpartum depression very often show comorbid anxiety (Farr et al., 2014). The main predictor for depressive, anxiety, or psychotic diseases after delivery is an antenatal episode of the illness (Perani and Slattery, 2014). Thus, WKY and WMI females do have risk factors for showing characteristics of postpartum depression after delivery.

The decreased self-directed behavior of the WMI dams became very apparent in this study because we observed maternal behavior during both the light and the dark phase for 10 days postpartum. This is in contrast to some other studies in which spot check observations were conducted on PPD 4 and 9 only (Braw et al., 2009), or behaviors were examined on PPD 3–4 and 17–18 only in the light phase (Lavi-Avnon et al., 2005). Nocturnal animals eat and drink more during the dark, just as the WLI dams did, but the disturbed rhythm of WMIs recalls a similar phenomenon in the WKYs. The WKY rats were found to be less responsive to light, which may cause possible alterations in daily rhythm patterns of this strain (Rosenwasser, 1993; Solberg et al., 2001). Interestingly, the FSL has shown a similar phenotype, suggesting that it may be a common phenotype in animal models of depression (Shiromani and Overstreet, 1994). Some data demonstrate that patients with depression may have an altered sensitivity to light (Duncan, 1996). Additionally, greater biological rhythm disturbances have been observed in patients with depression compared to healthy controls (Mondin et al., 2017; Ozcelik and Sahbaz, 2020). Sleep disturbances have also been observed in WKYs (Dugovic et al., 2000; Dasilva et al., 2011), very similar to those described in depressed patients (Rosenwasser and Wirz-Justice, 1997). Thus, the lack of diurnal rhythm of self-directed behaviors in the WMI dams suggests that this phenotype segregated with the depression-like behavior.

Because the WLI and WMI strains are isogenic, we could peruse the sequences for possible causative sequence variations that may be associated with the lack of diurnal rhythm observed in the WMI dams. The confirmed nonsynonymous sequence variation in the coding region of lysine-specific demethylase 5A (*Kdm5a*/*Jarid1a*) elevated this gene to a possible causative gene, although the single nucleotide polymorphism (SNP) was in the WLI strain. Kdm5a forms a complex with transcription factors Clock and Bmal1, which results in transcriptional activation of the Period genes and maintenance of circadian oscillations (DiTacchio et al., 2011). Interestingly, *Kdm5a* and *Per2* expression were increased in the WMI compared to WLI, regardless of litter survival or postpartum days. Increased hypothalamic expression of *Kdm5a* and *Per2* may contribute to the lack of circadian rhythm seen in the WMI’s behavior. Constitutive expression of *Per2* abolishes diurnal rhythm (Chen et al., 2009), and *Kdm5a* is known to activate *Per2* expression (DiTacchio et al., 2011). Thus, the lack of diurnal variation in self-directed behaviors of the WMI dam could be related to the increased expression of *Per2* in the hypothalamus. Whether the increased *Per2* is also a molecular characterization of the WMI’s depressive behavior is not known, but antidepressants are known to reduce *Per2* expression in experimental models (Orozco-Solis et al., 2017).

Alternatively, the SNP in the *Kdm5a* gene in the WLIs could have a loss or gain of function effect. The gain of function could enhance the circadian rhythm of WLI in their self-directed behavior, and the Kdm5a-induced negative regulation of transcription by RNA polymerase II could suppress the expression of the target genes in WLIs, and not in WMIs. However, there were no correlations between the expression of *Kdm5a* and other genes except for *Per2* in the WLIs. In contrast, *Kdm5a* expression correlated with *Avp*, but only in the WMIs, suggesting a loss of function effect of the WLI SNP. These proposed mechanisms do not explain the lack of diurnal rhythm in the WMIs self-directed behavior, as no single regulator can. However, they implicate that genetic manipulations in these strains could, potentially, identify causative variations affecting maternal behavior.

The observed increases in the hypothalamic expression of *Oxtr*, *Avp*, and *Avp1ra* in the WMI dams compared to WLIs and also to those WMIs whose litter died are difficult to interpret in the context of their accepted role in maternal behavior. A large body of literature supports the role of neuroendocrine processes in the induction and regulation of maternal behavior (Bridges, 2015). Both neuropeptides, Oxt and Avp, have been known to have an impact on maternal behavior (Pedersen and Prange, 1979; Pedersen et al., 1982; Bosch and Neumann, 2008; Bayerl and Bosch, 2019). Their receptors, Avpr1a and Oxtr, have also been implicated in maternal behaviors (Donaldson and Young, 2008) with complementary expression patterns in the ventromedial hypothalamus (Raggenbass, 2008). Several rat and mice studies showed a correlation between *Oxtr* and *Avpr* expression, similar to what is seen in the WLI dams’ hypothalami. However, more maternal care has been associated with higher levels of oxytocin binding in relevant brain regions (Champagne et al., 2001; Curley et al., 2012), including the paraventricular nucleus of the hypothalamus (Bayerl et al., 2016). Furthermore, the central administration of an Oxtr antagonist reduces high levels of pup licking, grooming, and arched-back nursing in dams (Champagne et al., 2001). Thus, increased pup licking, grooming behavior of the WMI dam is in concordance with their increased hypothalamic expression of *Oxtr*, but not with their decreased arched-back and blanket nursing compared to that of WLIs. Administration of Avp increases pup grooming in rats (Caldwell et al., 1986; Elkabir et al., 1990), while antagonism of Avpr1a does not affect arched back nursing and pup retrieval, but decreases other types of nursing (Bayerl et al., 2016). Thus again, the increased *Avp* expression in the WMI hypothalamus is as per a component of the licking, grooming behavior of WMI dams, but not with the rest of the observed behavior. These seeming contradictions suggest that discrete components of maternal behavior are influenced by different neuropeptidergic mechanisms or differing neurocircuitry (Bosch and Neumann, 2012). The complexity of the association between maternal behavioral differences and these neuropeptidergic mechanisms is exaggerated by the findings that the administration of an Avpr1a antagonist reduces anxiety/depression-like behavior in preclinical studies (Wigger et al., 2004). Furthermore, *AVP* and *AVPR1A* expressions are higher in the human hypothalamus of depressed patients compared to that of controls (Meynen et al., 2006; Wang et al., 2008). Therefore, the increased hypothalamic expression of *Avp* and *Avpr1a* could be the manifestation of the depression-like behavior of the WMI dams.

The most thought-provoking findings of the present study, such as the increased licking, grooming, and retrieval but decreased arched-back and blanket nursing and self-directed behaviors of the WMI dams, seem to parallel postpartum behavior of its progenitor, the WKY strain, which is considered an animal model of depression. In contrast, these behaviors are very different in the WLIs, where the inbred strain does not show depression-like behavior. Thus, some postpartum behaviors segregated together with depression-like behavior between the two strains during selective breeding. Genetic studies using the WKY and another strain with phenotypic differences are possible, but less likely to produce causative genes due to the nature of these quantitative trait loci studies (Solberg et al., 2004). In contrast, exploring the underlying genetic basis of these behaviors in the isogenic WLI and WMI strains using the reduced complexity cross approach would be more meaningful, as we described it recently (Bryant et al., 2020). The loss of diurnal patterns in self-directed behaviors and increased hypothalamic *Avp* and *Avpr1a* expression are resonant to findings in human depressed patients. Future studies could investigate whether attenuating the WMI’s depression-like behavior before gestation, by antidepressant treatment (Will et al., 2003) or by environmental enrichment (Mehta-Raghavan et al., 2016), would equalize litter survival, maternal and self-directed behaviors, and neuropeptide receptor expression in the hypothalamus of WMI and WLI dams. Since the greatest risk factor for postpartum depression is a history of depression before pregnancy (Putnam et al., 2017), the WLI and WMI rat strains could be valuable tools to investigate the molecular and genetic underpinning of this disorder.

## Data Availability Statement

The raw data supporting the conclusions of this article will be made available by the authors, without undue reservation.

## Ethics Statement

The animal study was reviewed and approved by Northwestern University Institutional ACUC.

## Author Contributions

The study was designed by EER and WL. Experimental work was carried out by WL, PHL, SLW, SAG, and HC. Data analysis was carried out by WL and EER. The manuscript was drafted by WL, EER, and HC. All authors contributed to the article and approved the submitted version.

## Conflict of Interest

The authors declare that the research was conducted in the absence of any commercial or financial relationships that could be construed as a potential conflict of interest.

## References

[B1] Ahmadiyeh N. Slone-Wilcoxon J. L. Takahashi J. S. Redei E. E. (2004). Maternal behavior modulates X-linked inheritance of behavioral coping in the defensive burying test. Biol. Psychiatry 55, 1069–1074. 10.1016/j.biopsych.2004.02.014 15158425 PMC3760164

[B3] Andrus B. M. Blizinsky K. Vedell P. T. Dennis K. Shukla P. K. Schaffer D. J.. (2012). Gene expression patterns in the hippocampus and amygdala of endogenous depression and chronic stress models. Mol. Psychiatry 17, 49–61. 10.1038/mp.2010.119 21079605 PMC3117129

[B4] Baum A. E. Solberg L. C. Churchill G. A. Ahmadiyeh N. Takahashi J. S. Redei E. E. (2006). Test- and behavior-specific genetic factors affect WKY hypoactivity in tests of emotionality. Behav. Brain Res. 169, 220–230. 10.1016/j.bbr.2006.01.007 16490266 PMC3762875

[B5] Bayerl D. S. Bosch O. J. (2019). Brain vasopressin signaling modulates aspects of maternal behavior in lactating rats. Genes Brain Behav. 18:e12517. 10.1111/gbb.12517 30221458

[B6] Bayerl D. S. Hönig J. N. Bosch O. J. (2016). Vasopressin V1a, but not V1b, receptors within the PVN of lactating rats mediate maternal care and anxiety-related behaviour. Behav. Brain Res. 305, 18–22. 10.1016/j.bbr.2016.02.020 26909846

[B9] Bosch O. J. Müsch W. Bredewold R. Slattery D. A. Neumann I. D. (2007). Prenatal stress increases HPA axis activity and impairs maternal care in lactating female offspring: implications for postpartum mood disorder. Psychoneuroendocrinology 32, 267–278. 10.1016/j.psyneuen.2006.12.012 17337328

[B7] Bosch O. J. Neumann I. D. (2008). Brain vasopressin is an important regulator of maternal behavior independent of dams’ trait anxiety. Proc. Natl. Acad. Sci. U S A 105, 17139–17144. 10.1073/pnas.0807412105 18955705 PMC2579391

[B8] Bosch O. J. Neumann I. D. (2012). Both oxytocin and vasopressin are mediators of maternal care and aggression in rodents: from central release to sites of action. Horm. Behav. 61, 293–303. 10.1016/j.yhbeh.2011.11.002 22100184

[B10] Bosch O. J. Pförtsch J. Beiderbeck D. I. Landgraf R. Neumann I. D. (2010). Maternal behaviour is associated with vasopressin release in the medial preoptic area and bed nucleus of the stria terminalis in the rat. J. Neuroendocrinol. 22, 420–429. 10.1111/j.1365-2826.2010.01984.x 20163514

[B11] Braw Y. Malkesman O. Merenlender A. Dagan M. Bercovich A. Lavi-Avnon Y.. (2009). Divergent maternal behavioral patterns in two genetic animal models of depression. Physiol. Behav. 96, 209–217. 10.1016/j.physbeh.2008.10.002 18957302

[B12] Bridges R. S. (2015). Neuroendocrine regulation of maternal behavior. Front. Neuroendocrinol. 36, 178–196. 10.1016/j.yfrne.2014.11.007 25500107 PMC4342279

[B13] Bryant C. D. Smith D. J. Kantak K. M. Nowak T. S. Jr. Williams R. W. Damaj M. I.. (2020). Facilitating complex trait analysis *via* reduced complexity crosses. Trends Genet. 36, 549–562. 10.1016/j.tig.2020.05.003 32482413 PMC7365571

[B14] Caldwell J. D. Drago F. Prange A. J. Jr. Pedersen C. A. (1986). A comparison of grooming behavior potencies of neurohypophyseal nonapeptides. Regul. Pept. 14, 261–271. 10.1016/0167-0115(86)90009-1 3014615

[B15] Champagne F. Diorio J. Sharma S. Meaney M. J. (2001). Naturally occurring variations in maternal behavior in the rat are associated with differences in estrogen-inducible central oxytocin receptors. Proc. Natl. Acad. Sci. U S A 98, 12736–12741. 10.1073/pnas.221224598 11606726 PMC60123

[B16] Champagne F. A. Francis D. D. Mar A. Meaney M. J. (2003). Variations in maternal care in the rat as a mediating influence for the effects of environment on development. Physiol. Behav. 79, 359–371. 10.1016/s0031-9384(03)00149-5 12954431

[B17] Chen H. Guryev V. Mulligan M. Redei E. Williams R. (2017). “Sequence variations between a genetic rat model of depression and its control strain,” in Proceedings of the 15th Annual Meeting of the Complex Trait Community in Collaboration with the 10th Annual Meeting of Rat Genomics and Models, Memphis, TN, USA.

[B18] Chen R. Schirmer A. Lee Y. Lee H. Kumar V. Yoo S.-H.. (2009). Rhythmic PER abundance defines a critical nodal point for negative feedback within the circadian clock mechanism. Mol. Cell 36, 417–430. 10.1016/j.molcel.2009.10.012 19917250 PMC3625733

[B19] Curley J. P. Jensen C. L. Franks B. Champagne F. A. (2012). Variation in maternal and anxiety-like behavior associated with discrete patterns of oxytocin and vasopressin 1a receptor density in the lateral septum. Horm. Behav. 61, 454–461. 10.1016/j.yhbeh.2012.01.013 22300676 PMC3312967

[B20] Dasilva J. K. Lei Y. Madan V. Mann G. L. Ross R. J. Tejani-Butt S.. (2011). Fear conditioning fragments REM sleep in stress-sensitive Wistar-Kyoto, but not Wistar, rats. Prog. Neuropsychopharmacol. Biol. Psychiatry 35, 67–73. 10.1016/j.pnpbp.2010.08.023 20832443 PMC3019280

[B21] DiTacchio L. Le H. D. Vollmers C. Hatori M. Witcher M. Secombe J.. (2011). Histone lysine demethylase JARID1a activates CLOCK-BMAL1 and influences the circadian clock. Science 333, 1881–1885. 10.1126/science.1206022 21960634 PMC3204309

[B22] Donaldson Z. R. Young L. J. (2008). Oxytocin, vasopressin and the neurogenetics of sociality. Science 322, 900–904. 10.1126/science.1158668 18988842

[B23] Dugovic C. Solberg L. C. Redei E. Reeth O. V. Turek F. W. (2000). Sleep in the Wistar-Kyoto rat, a putative genetic animal model for depression. Neuroreport 11, 627–631. 10.1097/00001756-200002280-00038 10718326

[B24] Duncan W. C. Jr. (1996). Circadian rhythms and the pharmacology of affective illness. Pharmacol. Ther. 71, 253–312. 10.1016/s0163-7258(96)00092-7 8940745

[B25] Eid R. S. Gobinath A. R. Galea L. A. M. (2019). Sex differences in depression: insights from clinical and preclinical studies. Prog. Neurobiol. 176, 86–102. 10.1016/j.pneurobio.2019.01.006 30721749

[B26] Elkabir D. R. Wyatt M. E. Vellucci S. V. Herbert J. (1990). The effects of separate or combined infusions of corticotrophin-releasing factor and vasopressin either intraventricularly or into the amygdala on aggressive and investigative behaviour in the rat. Regul. Pept. 28, 199–214. 10.1023/a:1012360809183 2343163

[B27] Falowski S. M. Sharan A. Reyes B. A. Sikkema C. Szot P. Van Bockstaele E. J. (2011). An evaluation of neuroplasticity and behavior after deep brain stimulation of the nucleus accumbens in an animal model of depression. Neurosurgery 69, 1281–1290. 10.1227/NEU.0b013e3182237346 21566538 PMC4707959

[B28] Fang Y. Y. Yamaguchi T. Song S. C. Tritsch N. X. Lin D. (2018). A hypothalamic midbrain pathway essential for driving maternal behaviors. Neuron 98, 192.e10–207.e10. 10.1016/j.neuron.2018.02.019 29621487 PMC5890946

[B29] Farr S. L. Dietz P. M. O’Hara M. W. Burley K. Ko J. Y. (2014). Postpartum anxiety and comorbid depression in a population-based sample of women. J. Womens Health 23, 120–128. 10.1089/jwh.2013.4438 24160774 PMC7469256

[B30] Francis D. D. Szegda K. Campbell G. Martin W. D. Insel T. R. (2003). Epigenetic sources of behavioral differences in mice. Nat. Neurosci. 6, 445–446. 10.1038/nn1038 12665797

[B32] Gill Iii T. Kunz H. Hansen C. (1979). Litter sizes in inbred strains of rats (Rattus norvegicus). Int. J. Immunogenet. 6, 461–463. 10.1111/j.1744-313x.1979.tb00701.x 574888

[B33] Glynn L. M. Davis E. P. Sandman C. A. Goldberg W. A. (2016). Gestational hormone profiles predict human maternal behavior at 1-year postpartum. Horm. Behav. 85, 19–25. 10.1016/j.yhbeh.2016.07.002 27427279 PMC5929113

[B34] Grant K. A. Mcmahon C. Austin M. P. (2008). Maternal anxiety during the transition to parenthood: a prospective study. J. Affect. Disord. 108, 101–111. 10.1016/j.jad.2007.10.002 18001841

[B35] Hakanen H. Flykt M. Sinervä E. Nolvi S. Kataja E.-L. Pelto J.. (2019). How maternal pre- and postnatal symptoms of depression and anxiety affect early mother-infant interaction? J. Affect. Disord. 257, 83–90. 10.1016/j.jad.2019.06.048 31299408

[B36] Hauser H. Gandelman R. (1985). Lever pressing for pups: evidence for hormonal influence upon maternal behavior of mice. Horm. Behav. 19, 454–468. 10.1016/0018-506x(85)90041-8 4085998

[B37] Jeannotte A. M. Mccarthy J. G. Redei E. E. Sidhu A. (2008). Desipramine modulation of α-, γ-synuclein and the norepinephrine transporter in an animal model of depression. Neuropsychopharmacology 34, 987–998. 10.1038/npp.2008.146 18800064

[B38] Jensen Peña C. Champagne F. A. (2013). Implications of temporal variation in maternal care for the prediction of neurobiological and behavioral outcomes in offspring. Behav. Neurosci. 127, 33–46. 10.1037/a0031219 23398440 PMC3947603

[B39] Jordan S. Davies G. I. Thayer D. S. Tucker D. Humphreys I. (2019). Antidepressant prescriptions, discontinuation, depression and perinatal outcomes, including breastfeeding: a population cohort analysis. PLoS One 14:e0225133. 10.1371/journal.pone.0225133 31738813 PMC6860440

[B40] Kalsbeek A. Buijs R. M. (2002). Output pathways of the mammalian suprachiasmatic nucleus: coding circadian time by transmitter selection and specific targeting. Cell Tissue Res. 309, 109–118. 10.1007/s00441-002-0577-0 12111541

[B41] Kokras N. Pastromas N. Papasava D. De Bournonville C. Cornil C. A. Dalla C. (2018). Sex differences in behavioral and neurochemical effects of gonadectomy and aromatase inhibition in rats. Psychoneuroendocrinology 87, 93–107. 10.1016/j.psyneuen.2017.10.007 29054014

[B42] Kurtz T. W. Montano M. Chan L. Kabra P. (1989). Molecular evidence of genetic heterogeneity in Wistar-Kyoto rats: implications for research with the spontaneously hypertensive rat. Hypertension 13, 188–192. 10.1161/01.hyp.13.2.188 2914738

[B43] Kyeremanteng C. James J. Mackay J. Merali Z. (2012). A study of brain and serum brain-derived neurotrophic factor protein in Wistar and Wistar-Kyoto rat strains after electroconvulsive stimulus. Pharmacopsychiatry 45, 244–249. 10.1055/s-0032-1306278 22454252

[B44] Lavi-Avnon Y. Shayit M. Yadid G. Overstreet H. D. Weller A. (2005). Immobility in the swim test and observations of maternal behavior in lactating Flinders sensitive line rats. Behav. Brain Res. 161, 155–163. 10.1016/j.bbr.2005.02.002 15904722

[B45] Leng G. Meddle S. L. Douglas A. J. (2008). Oxytocin and the maternal brain. Curr. Opin. Pharmacol. 8, 731–734. 10.1016/j.coph.2008.07.001 18656552

[B46] Levy F. (2016). Neuroendocrine control of maternal behavior in non-human and human mammals. Ann. Endocrinol. 77, 114–125. 10.1016/j.ando.2016.04.002 27130073

[B48] Lim P. H. Shi G. Wang T. Jenz S. T. Mulligan M. K. Redei E. E.. (2018a). Genetic model to study the co-morbid phenotypes of increased alcohol intake and prior stress-induced enhanced fear memory. Front. Genet. 9:566. 10.3389/fgene.2018.00566 30538720 PMC6277590

[B49] Lim P. H. Wert S. L. Tunc-Ozcan E. Marr R. Ferreira A. Redei E. E. (2018b). Premature hippocampus-dependent memory decline in middle-aged females of a genetic rat model of depression. Behav. Brain Res. 353, 242–249. 10.1016/j.bbr.2018.02.030 29490235

[B50] Louis W. J. Howes L. G. (1990). Geneaology of the spontaneously hypertensive rat and Wistar-kyoto rat strains: implications for studies of inherited hypertension. J. Cardiovasc. Pharmacol. 16, S1–S5. 1708002

[B51] Lynch A. (1976). Postnatal undernutrition: an alternative method. Dev. Psychobiol. 9, 39–48. 10.1002/dev.420090107 943351

[B52] Malkesman O. Braw Y. Zagoory-Sharon O. Golan O. Lavi-Avnon Y. Schroeder M.. (2005). Reward and anxiety in genetic animal models of childhood depression. Behav. Brain Res. 164, 1–10. 10.1016/j.bbr.2005.04.023 16055204

[B53] Masri S. Sassone-Corsi P. (2013). The circadian clock: a framework linking metabolism, epigenetics and neuronal function. Nat. Rev. Neurosci. 14:69. 10.1038/nrn3393 23187814 PMC5720680

[B54] Meckes J. K. Lim P. H. Wert S. L. Luo W. Gacek S. A. Platt D.. (2018). Brain region-specific expression of genes mapped within quantitative trait loci for behavioral responsiveness to acute stress in Fisher 344 and Wistar Kyoto male rats. PLoS One 13:e0194293. 10.1371/journal.pone.0194293 29529077 PMC5847310

[B55] Mehta N. S. Wang L. Redei E. E. (2013). Sex differences in depressive, anxious behaviors and hippocampal transcript levels in a genetic rat model. Genes Brain Behav. 12, 695–704. 10.1111/gbb.12063 23876038

[B56] Mehta-Raghavan N. S. Wert S. L. Morley C. Graf E. N. Redei E. E. (2016). Nature and nurture: environmental influences on a genetic rat model of depression. Transl. Psychiatry 6:e770. 10.1038/tp.2016.28 27023176 PMC4872452

[B57] Meynen G. Unmehopa U. A. Van Heerikhuize J. J. Hofman M. A. Swaab D. F. Hoogendijk W. J. (2006). Increased arginine vasopressin mRNA expression in the human hypothalamus in depression: a preliminary report. Biol. Psychiatry 60, 892–895. 10.1016/j.biopsych.2005.12.010 16499879

[B58] Mondin T. C. Cardoso T. A. Souza L. D. M. Jansen K. Da Silva Magalhães P. V. Kapczinski F.. (2017). Mood disorders and biological rhythms in young adults: a large population-based study. J. Psychiatr. Res. 84, 98–104. 10.1016/j.jpsychires.2016.09.030 27716514

[B59] Murakami G. (2016). Distinct effects of estrogen on mouse maternal behavior: the contribution of estrogen synthesis in the brain. PLoS One 11:e0150728. 10.1371/journal.pone.0150728 27007402 PMC4805179

[B60] Myers M. M. Brunelli S. A. Shair H. N. Squire J. M. Hofer M. A. (1989). Relationships between maternal behavior of SHR and WKY dams and adult blood pressures of cross-fostered F1 pups. Dev. Psychobiol. 22, 55–67. 10.1002/dev.420220105 2912813

[B61] Orozco-Solis R. Montellier E. Aguilar-Arnal L. Sato S. Vawter M. P. Bunney B. G.. (2017). A circadian genomic signature common to ketamine and sleep deprivation in the anterior cingulate cortex. Biol. Psychiatry 82, 351–360. 10.1016/j.biopsych.2017.02.1176 28395871 PMC5660920

[B62] Ozcelik M. Sahbaz C. (2020). Clinical evaluation of biological rhythm domains in patients with major depression. Braz. J. Psychiatry 42, 258–263. 10.1590/1516-4446-2019-0570 32022159 PMC7236150

[B63] Paré W. P. (1994a). Hyponeophagia in Wistar Kyoto (WKY) rats. Physiol. Behav. 55, 975–978. 10.1016/0031-9384(94)90090-6 8022922

[B64] Paré W. P. (1994b). Open field, learned helplessness, conditioned defensive burying and forced-swim tests in WKY rats. Physiol. Behav. 55, 433–439. 10.1016/0031-9384(94)90097-3 8190758

[B65] Paré W. P. Kluczynski J. (1997). Differences in the stress response of Wistar-Kyoto (WKY) rats from different vendors. Physiol. Behav. 62, 643–648. 10.1016/s0031-9384(97)00191-1 9272677

[B66] Paré W. P. Redei E. (1993). Depressive behavior and stress ulcer in Wistar Kyoto rats. J. Physiol. 87, 229–238. 10.1016/0928-4257(93)90010-q 8136789

[B67] Parfitt Y. Pike A. Ayers S. (2013). The impact of parents’ mental health on parent-baby interaction: a prospective study. Infant Behav. Dev. 36, 599–608. 10.1016/j.infbeh.2013.06.003 23850989

[B70] Pedersen C. A. Ascher J. A. Monroe Y. L. Prange A. J. Jr. (1982). Oxytocin induces maternal behavior in virgin female rats. Science 216, 648–650. 10.1126/science.7071605 7071605

[B68] Pedersen C. A. Boccia M. L. (2003). Oxytocin antagonism alters rat dams’ oral grooming and upright posturing over pups. Physiol. Behav. 80, 233–241. 10.1016/j.physbeh.2003.07.011 14637221

[B71] Pedersen C. A. Caldwell J. D. Walker C. Ayers G. Mason G. A. (1994). Oxytocin activates the postpartum onset of rat maternal behavior in the ventral tegmental and medial preoptic areas. Behav. Neurosci. 108, 1163–1171. 10.1037/0735-7044.108.6.1163 7893408

[B69] Pedersen C. A. Prange A. J. Jr. (1979). Induction of maternal behavior in virgin rats after intracerebroventricular administration of oxytocin. Proc. Natl. Acad. Sci. U S A 76, 6661–6665. 10.1073/pnas.76.12.6661 293752 PMC411928

[B72] Perani C. Slattery D. (2014). Using animal models to study post-partum psychiatric disorders. Br. J. Pharmacol. 171, 4539–4555. 10.1111/bph.12640 24527704 PMC4209931

[B73] Pratt M. Apter-Levi Y. Vakart A. Feldman M. Fishman R. Feldman T.. (2015). Maternal depression and child oxytocin response; moderation by maternal oxytocin and relational behavior. Depress Anxiety 32, 635–646. 10.1002/da.22392 26130435

[B74] Putnam K. T. Wilcox M. Robertson-Blackmore E. Sharkey K. Bergink V. Munk-Olsen T.. (2017). Clinical phenotypes of perinatal depression and time of symptom onset: analysis of data from an international consortium. Lancet Psychiatry 4, 477–485. 10.1016/S2215-0366(17)30136-0 28476427 PMC5836292

[B75] Raggenbass M. (2008). Overview of cellular electrophysiological actions of vasopressin. Eur. J. Pharmacol. 583, 243–254. 10.1016/j.ejphar.2007.11.074 18280467

[B76] Raghavan N. S. Chen H. Schipma M. Luo W. Chung S. Wang L.. (2017). Prepubertal ovariectomy exaggerates adult affective behaviors and alters the hippocampal transcriptome in a genetic rat model of depression. Front. Endocrinol. 8:373. 10.3389/fendo.2017.00373 29403433 PMC5786888

[B77] Redei E. E. Solberg L. C. Kluczynski J. M. Pare W. P. (2001). Paradoxical hormonal and behavioral responses to hypothyroid and hyperthyroid states in the Wistar-Kyoto rat. Neuropsychopharmacology 24, 632–639. 10.1016/S0893-133X(00)00229-3 11331143

[B78] Rich-Edwards J. W. Kleinman K. Abrams A. Harlow B. L. Mclaughlin T. J. Joffe H.. (2006). Sociodemographic predictors of antenatal and postpartum depressive symptoms among women in a medical group practice. J. Epidemiol. Community Health 60, 221–227. 10.1136/jech.2005.039370 16476752 PMC2465548

[B79] Rosenwasser A. M. (1993). Circadian drinking rhythms in SHR and WKY rats: effects of increasing light intensity. Physiol. Behav. 53, 1035–1041. 10.1016/0031-9384(93)90356-k 8346285

[B2] Rosenwasser A. M. Wirz-Justice A. (1997). “Circadian rhythms and depression: clinical and experimental models,” in Physiology and Pharmacology of Biological Rhythms, eds Redfern P. H. Lemmer B. (Berlin: Springer), 457–486.

[B80] Shiromani P. J. Overstreet D. (1994). Free-running period of circadian rhythms is shorter in rats with a genetically upregulated central cholinergic system. Biol. Psychiatry 36, 622–626. 10.1016/0006-3223(94)90075-2 7833429

[B81] Solberg L. C. Ahmadiyeh N. Baum A. E. Vitaterna M. H. Takahashi J. S. Turek F. W.. (2003). Depressive-like behavior and stress reactivity are independent traits in a Wistar Kyoto × Fisher 344 cross. Mol. Psychiatry 8, 423–433. 10.1038/sj.mp.4001255 12740600

[B82] Solberg L. C. Baum A. E. Ahmadiyeh N. Shimomura K. Li R. Turek F. W.. (2004). Sex- and lineage-specific inheritance of depression-like behavior in the rat. Mamm. Genome 15, 648–662. 10.1007/s00335-004-2326-z 15457344 PMC3764448

[B83] Solberg L. C. Olson S. L. Turek F. W. Redei E. (2001). Altered hormone levels and circadian rhythm of activity in the WKY rat, a putative animal model of depression. Am. J. Physiol. Regul. Integr. Comp. Physiol. 281, R786–R794. 10.1152/ajpregu.2001.281.3.R786 11506993

[B84] Stein A. Craske M. G. Lehtonen A. Harvey A. Savage-Mcglynn E. Davies B.. (2012). Maternal cognitions and mother-infant interaction in postnatal depression and generalized anxiety disorder. J. Abnorm. Psychol. 121, 795–809. 10.1037/a0026847 22288906 PMC3506203

[B85] Topiwala A. Hothi G. Ebmeier K. P. (2012). Identifying patients at risk of perinatal mood disorders. Practitioner 256, 15–18. 22774377

[B86] Wallenborn J. T. Joseph A.-C. Graves W. C. Masho S. W. (2018). Prepregnancy depression and breastfeeding duration: a look at maternal age. J. Pregnancy 2018:4825727. 10.1155/2018/4825727 30515328 PMC6236915

[B87] Wang S. S. Kamphuis W. Huitinga I. Zhou J. N. Swaab D. F. (2008). Gene expression analysis in the human hypothalamus in depression by laser microdissection and real-time PCR: the presence of multiple receptor imbalances. Mol. Psychiatry 13, 786–799, 741. 10.1038/mp.2008.38 18427561

[B88] Weber E. M. Hultgren J. Algers B. Olsson I. A. S. (2016). Do laboratory mouse females that lose their litters behave differently around parturition? PLoS One 11:e0161238. 10.1371/journal.pone.0161238 27575720 PMC5005013

[B89] Wigger A. Sánchez M. M. Mathys K. C. Ebner K. Frank E. Liu D.. (2004). Alterations in central neuropeptide expression, release and receptor binding in rats bred for high anxiety: critical role of vasopressin. Neuropsychopharmacology 29, 1–14. 10.1038/sj.npp.1300290 12942143

[B90] Wilcoxon J. S. Kuo A. G. Disterhoft J. F. Redei E. E. (2005). Behavioral deficits associated with fetal alcohol exposure are reversed by prenatal thyroid hormone treatment: a role for maternal thyroid hormone deficiency in FAE. Mol. Psychiatry 10, 961–971. 10.1038/sj.mp.4001694 15940294

[B91] Will C. C. Aird F. Redei E. E. (2003). Selectively bred Wistar-Kyoto rats: an animal model of depression and hyper-responsiveness to antidepressants. Mol. Psychiatry 8, 925–932. 10.1038/sj.mp.4001345 14593430

[B92] Wisner K. L. Sit D. K. Moses-Kolko E. L. Driscoll K. E. Prairie B. Stika C. S.. (2015). Transdermal estradiol treatment for postpartum depression: a pilot randomized trial. J. Clin. Psychopharmacol. 35, 389–395. 10.1097/JCP.0000000000000351 26061609 PMC4485597

