# Supplementary material for: Hypothalamic Gene Expression and Postpartum Behavior in a Genetic Rat Model of Depression
Source: Front Behav Neurosci. 2020 Oct 22;14:589967. doi: 10.3389/fnbeh.2020.589967 (PMC7649805; doi:10.3389/fnbeh.2020.589967)
Supplement: Supplementary file 1 [file Table_1.DOCX]

**Hypothalamic gene expression and postpartum behavior in a genetic rat model of depression**

W Luo^1^, PH Lim^1^, SL Wert^1^, SA Gacek^1^, H Chen^2^, and E. E. Redei^1*^

Supplementary Material

**Supplementary Figure S1.** Sanger sequencing confirms a missense SNP between the WMI and WLI strains in the *Kdm5a* gene located on chr4:152938803. The C-T mutation (WMI is the same as the reference genome) corresponds to Arg745Cys in the Kdm5a protein.

| WMI | WLI |
| --- | --- |
| 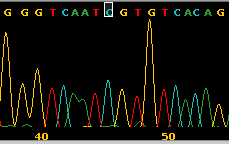 | 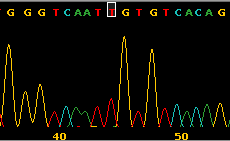 |

**Supplementary Figure S2**. Heatmaps of gene expression correlations in WLI and WMI dams whose litters died before postpartum day 5 (PPD <5) and in those whose litters were weaned at PPD 24. Positive correlations are shown in red for WLI dams and in blue for WMI dams. Negative correlations are the inverse of this, blue for WLIs and red for WMIs. Pearson r values are shown on the heatmap. Significant correlations (p < 0.05 after multiple comparisons) are indicated by a bolded square.


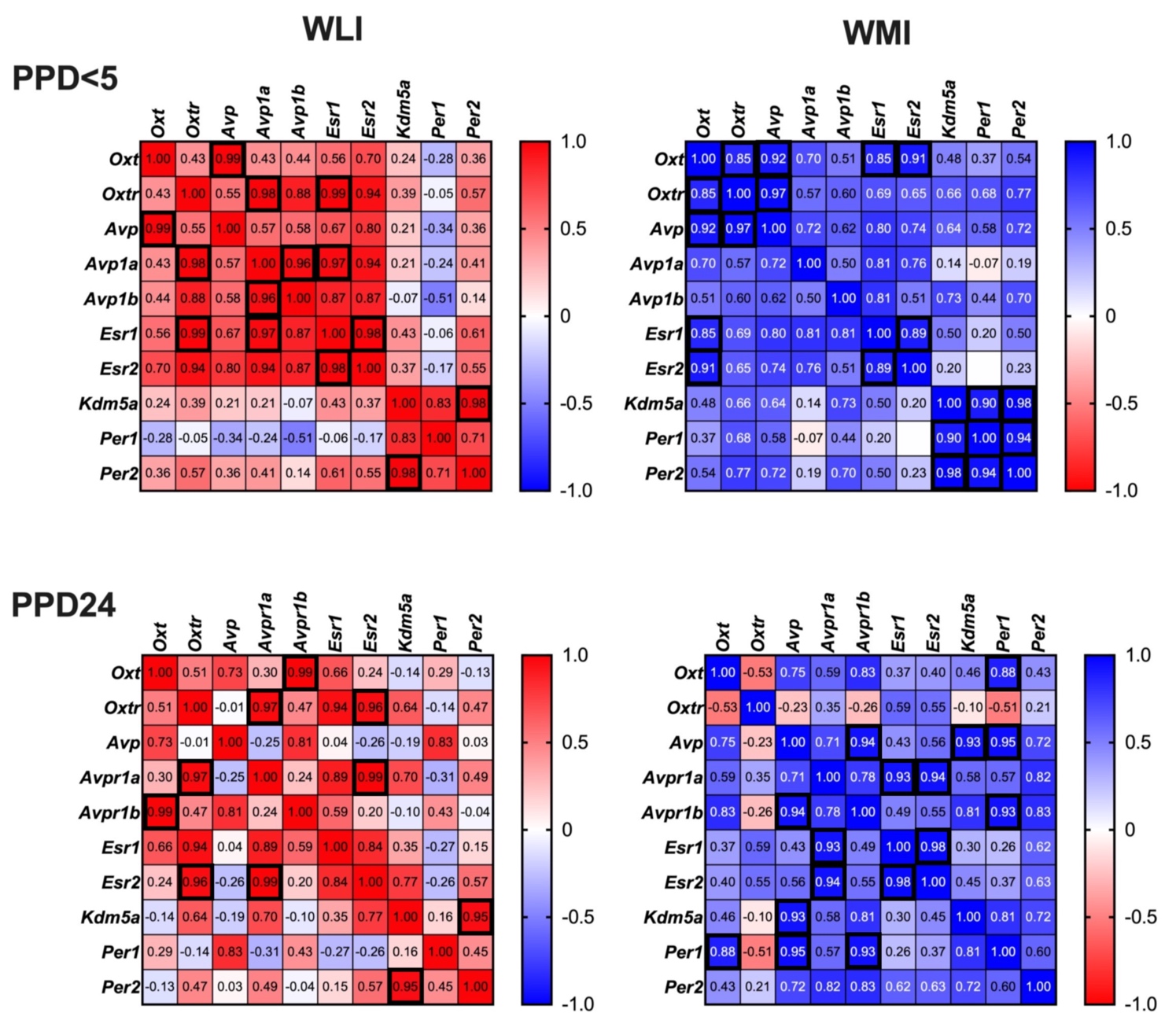


**Supplementary Table S1. Quantitative RT-PCR primer sequences. F- Forward; R- Reverse**

| Gene |  | Sequence 5’ - 3’ | Amplicon length (bp) |
| --- | --- | --- | --- |
| *Avp* | *F* | GAGAACTACCTGCCCTCGCC | 103 |
|  | *R* | CCACGCAGCTCTCATCGCT |  |
| *Avpr1a* | *F* | CTCCGATAGCATGAGCCGAA | 91 |
|  | *R* | AGATTTGGGCGAGTCCTTCC |  |
| *Avpr1b* | *F* | TCCAGGGCAAAGATCCGAAC | 115 |
|  | *R* | GGGCATTCTCATCCCACACA |  |
| *Esr1* | *F* | GAAAGGCGGGATACGAAAAGA | 59 |
|  | *R* | TCTGACGCTTGTGCTTCAACA |  |
| *Esr2* | *F* | CATCAGTAACAAGGGCATGGAA | 61 |
|  | *R* | CACCGGGACCACATTTTTG |  |
| *Gapdh* | *F* | CAACTCCCTCAAGATTGTCAGCAA | 118 |
|  | *R* | GGCATGGACTGTGGTCATGA |  |
| *Kdm5a* | *F* | CCCACTTGAAGACCTCCCAT | 84 |
|  | *R* | TCTGTGACACGATTGACCCA |  |
| *Oxt* | *F* | GTCTTGCTTGCTGCCTGCTTG | 115 |
|  | *R* | GGAAGACACTTGCGCATATCCA |  |
| *Oxtr* | *F* | CGTACTGGCCTTCATCGTGT | 71 |
|  | *R* | CATTGACGTCCCAAACGCTC |  |
| *Per1* | *F* | TATGCTCTGGCCTGTGTCAA | 120 |
|  | *R* | CTCCAATTCCTCCAGGGTGT |  |
| *Per2* | *F* | CAGTGCGTTCCCTTATGTGG | 185 |
|  | *R* | AACGTACAGTGTGGGTGCTA |  |
